# Evaluating a novel 3D printed model for simulating Large Loop Excision of the Transformation Zone (LLETZ)

**DOI:** 10.1186/s41205-022-00143-x

**Published:** 2022-06-08

**Authors:** Matthias Kiesel, Inga Beyers, Adam Kalisz, Achim Wöckel, Sanja Löb, Tanja Schlaiss, Christine Wulff, Joachim Diessner

**Affiliations:** 1 Department of Gynecology, University Hospital Würzburg, Josef-Schneider-Str. 4, 97080 Würzburg, Germany; 2 Institute of Electric Power Systems (IfES), Leibniz Universität Hannover, Appelstraße 9A, 30167 Hannover, Germany; 3 Department of Electrical, Electronic and Communication Engineering, Information Technology (LIKE), Friedrich-Alexander-Universität Erlangen-Nürnberg, Am Wolfsmantel 33, Erlangen, Germany

**Keywords:** 3D printing, Simulation, Gynecology, Loop electrosurgical excision procedure (LEEP), Large loop excision of the transformation zone (LLETZ), Teaching, Education, Patient safety, Cervical dysplasia

## Abstract

**Background:**

Electrosurgical excisions are common procedures for treating cervical dysplasia and are often seen as minor surgeries. Yet, thorough training of this intervention is required, as there are considerable consequences of inadequate resections, e.g. preterm birth, the risk of recurrence, injuries and many more. Unfortunately, there is a lack of sufficiently validated possibilities of simulating electrosurgeries, which focus on high fidelity and patient safety.

**Methods:**

A novel 3D printed simulator for examination and electrosurgical treatment of dysplastic areas of the cervix was compared with a conventional simulator. Sixty medical students experienced a seminar about cervical dysplasia. Group A underwent the seminar with the conventional and Group B with the novel simulator. After a theoretical introduction, the students were randomly assigned by picking a ticket from a box and went on to perform the hands-on training with their respective simulator. Each student first obtained colposcopic examination training. Then he or she performed five electrosurgical excisions (each). This was assessed with a validated score, to visualize their learning curve. Furthermore, adequate and inadequate resections and contacts between electrosurgical loop and vagina or speculum were counted. Both groups also assessed the seminar and their simulator with 18 questions (Likert-scales, 1–10, 1 = strongly agree / very good, 10 = strongly disagree / very bad). Group B additionally assessed the novel simulator with four questions (similar Likert-scales, 1–10).

**Results:**

Nine of 18 questions showed statistically significant differences favoring Group B (*p* < 0.05). Group B also achieved more adequate R0-resections and less contacts between electrosurgical loop and vagina or speculum. The learning curves of the performed resections favored the novel simulator of Group B without statistically significant differences. The four questions focusing on certain aspects of the novel simulator indicate high appreciation of the students with a mean score of 1.6 points.

**Conclusion:**

The presented novel simulator shows several advantages compared to the existing model. Thus, novice gynecologists can be supported with a higher quality of simulation to improve their training and thereby patient safety.

**Supplementary Information:**

The online version contains supplementary material available at 10.1186/s41205-022-00143-x.

## Background

Most cases of cervical cancer develop from dysplastic cells of the cervix [1, 2]. Cervical cancer itself is one of the most common malignancies of women with the fourth highest incidence and mortality of female cancers from a worldwide perspective, with estimated 604,000 new cases and 342,000 related deaths [3]. Precancerous lesions of the cervix affect approximately 1 % of all women [2]. Screening-methods such as Pap-smear can detect such lesions before progression into cervical cancer occurs [4]. If the Pap-smear indicates dysplastic changes, a trained colposcopist is required to examine the cervix. The examination is performed by applying acetic acid on the cervix under magnification, to make dysplastic areas visible. If signs of dysplasia are observed, a selective biopsy can be taken [5, 6]. If any precancerous lesions are confirmed, local excision in the form of LLETZ (Large Loop Excision of the Transformation Zone) or LEEP (Loop Electrosurgical Excision Procedure) is required [2, 7–14]. Both terms, LLETZ and LEEP, are often used synonymously. The term LLETZ can be applied for larger excisions, as it describes the entire removal of the transformation zone of the cervix [15]. Estimations concerning the number of performed LLETZ or LEEP vary greatly, reaching up to 140.000 excisions per in year in Germany [16].

On the one hand, adequate resection has to be achieved in cases of High grade Squamous Intraepithelial Lesion (HSIL), in order to correctly remove all of the dysplastic tissue [11–14, 17]. On the other hand, adverse events in future pregnancies, caused by inappropriate resection, must be avoided, as well as scarring of cervical tissue, which potentially leads to infertility [18–25]. Moreover, acute complications, such as vaginal injury and thermal damage of vagina, vulva or urethra can occur, especially if specula without adequate coating for electrical insulation are used. Even uterine perforation, bowel injury, intraabdominal bleeding and peritonitis have been reported [26–30]. Hence, LLETZ or LEEP require thorough and vigorous training before a novice surgeon performs his or her first surgery on a patient.

There are options to achieve sufficient simulation of electrosurgery of the cervix, in order to improve patient safety. Unfortunately, the framework for ample training often is difficult, and there is little scientific data [31–41]. Firstly, there is a lack of standardization for the simulation of LEEP or LLETZ. Secondly, such training comes with a considerable workload, which is challenging when taking into account the time and personnel constraints in everyday clinical work. Thirdly, the presented simulators for LLETZ or LEEP up until now mainly focus on three aspects: a) the basic steps of electrosurgery, b) quick assembly of simulation apparatuses and c) on low-budget solutions. Consequently, these phantoms consist of simple components, which was found to insufficiently represent the real female anatomy [31, 37, 40]. Fourthly, according to the authors’ knowledge, there has neither been a study comparing two simulators for LLETZ or LEEP yet, nor has a simulation considered possible complications during the procedure. Due to this, based on previous work describing the development and construction of a new high fidelity simulator [42], this novel simulator is evaluated. This is done by comparing it to a conventional model consisting of simple materials such as a drain pipe and insulation foam reassembling the vulva and vagina, as well as a sausage mimicking the cervix [42]. Thus, high quality training-options for gynecologic surgeons are offered, thereby improving patient safety.

## Methods

### Preparation and execution of the seminar

At the Department of Gynecology at the University Hospital of Würzburg, 60 medical students in their fifth and sixth year, who had never performed a LLETZ or a LEEP before, took part in a voluntary seminar about cervical dysplasia from December 2020 to April 2021 as a single center study. All students exceeded 18 years of age and gave their consent in voluntarily participating in this work. All gained information was anonymized. A certificate of non-objection was obtained from the Ethics Committee of the University Hospital Würzburg (application number 2020080401). The seminar took place in the outpatient clinic of the Department of Gynecology at the University Hospital of Würzburg and was organized as a one-on-one teaching with the same Gynecologist with over 4 years of specialization in colposcopy, in order to avoid inter-teacher-variability. The colposcope used for the training was the model 150 FC from the company ZEISS (Jena, Germany) and was connected to a digital camera, which allowed the teacher to supervise all the students’ steps on a separate monitor.

The duration of each training was approximately 1.5 hours and consisted of a theoretical introduction with Microsoft PowerPoint about the concerning female anatomy and physiology, the pathophysiology of the development of cervical dysplasia together with its diagnosis and treatment, information about the functionality, use and safety instructions concerning electrosurgery, definition of type one electrosurgical excision, data collection according to the LEEP-score following Takacs et al. [31, 32] as well as the equipment and workflow of an outpatient clinic specialized on vulvar, vaginal and cervical dysplasia. The PowerPoint presentation contained explanations and 2D images. Each student was then randomly assigned to Group A or B by picking a ticket from a box. Group A then underwent the training with the conventional simulator and Group B with the novel simulator.

### The simulators

**Fig. 1:**
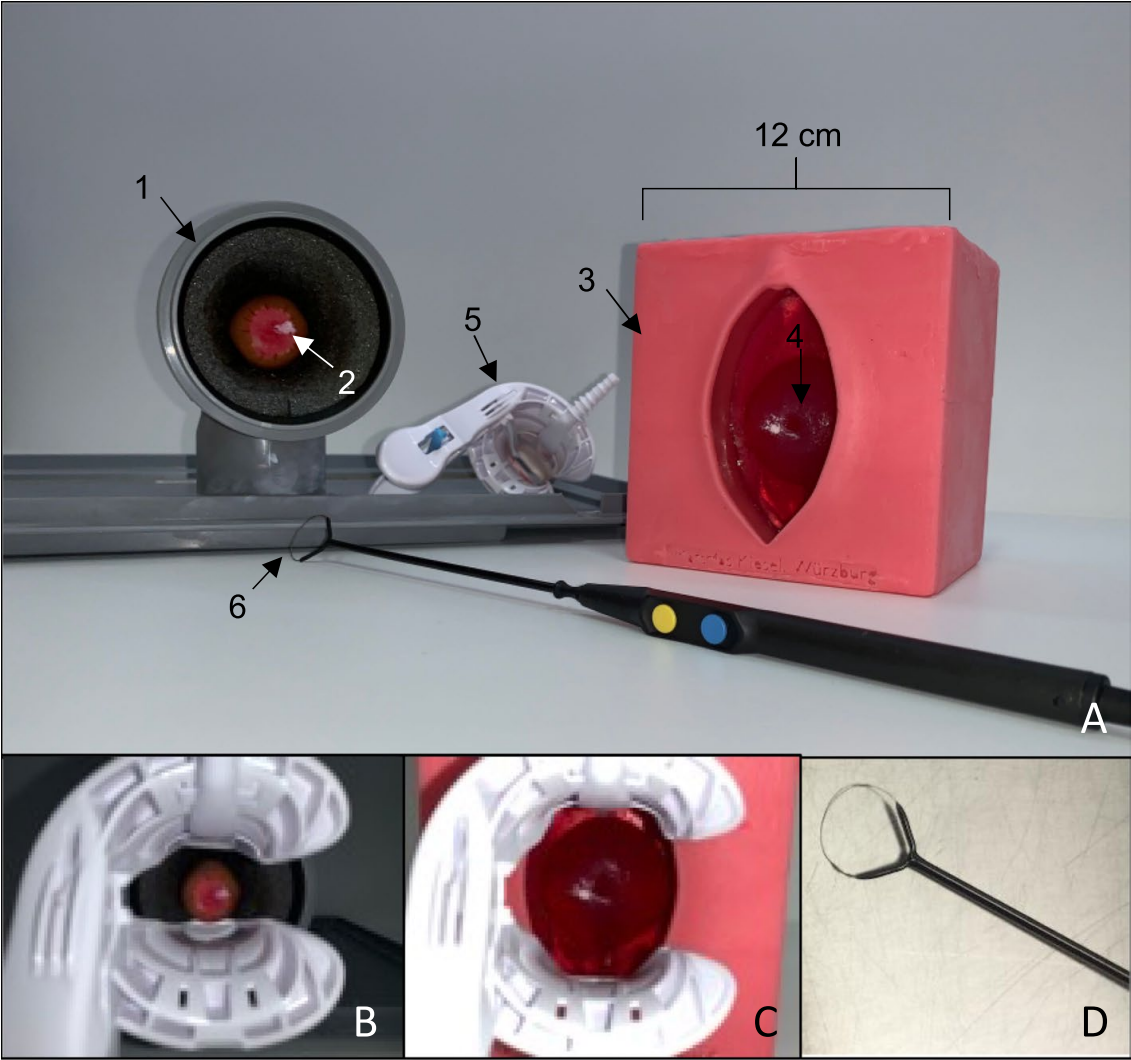
Depiction of the conventional and the novel simulator together with a speculum (Orchid Wide SX, Bridea Medical, Amsterdam, Netherlands) and electrosurgical loop (Erbe Elektromedizin GmbH, Tübingen, Germany). A: Both simulators and equipment, A1: Drain pipe of conventional simulator, A2: artificial cervix (sausage) of conventional simulator with acetic white stain, A3: novel simulator, A4: artificial cervix (modified algae powder) of novel simulator with acetic white stain and cervical os, A5: speculum, A6: electrosurgical loop, B: Speculum inserted into conventional simulator, C: Speculum inserted into novel simulator, D: electrosurgical loop

The conventional simulator was built according to the findings of Takacs et al., Connor et al. and Walters et al. and is composed of a sausage, simulating the cervix, placed into a drain pipe fitted with insulation material simulating the vulva and the vagina [31, 32, 39, 43]. The novel simulator was first created as virtual model with an open-source program for 3D modeling (*Blender*, version 2.82). Single parts were then 3D printed with material extrusion by using the *Ultimaker 2+ (*Utimaker BV, Utrecht, Netherlands*)* with Polyactide (PLA) of 2.85 mm diameter (DAS FILAMENT, Emskirchen, Germany). Silicone was utilized to duplicate the printed vulva and vagina, in order to reassemble realistic tissue. Modified algae-powder came to use for duplicating the 3D printed cervix by using casting methods. Conventional and novel simulator have been described and compared in theory in previous work [42]. In this work the production costs of the novel simulator were calculated with 263,11 EUR. The production costs for the conventional simulator were approximately 50,00 EUR, but vary according to the used materials. Yet, the running costs for the novel simulator were less expensive, as the purchased algae-powder for one novel cervix had a price of 33 cents. The sausages utilized for the conventional simulator had a price of 1 € each [42]. Figure 1 displays both simulators with their equipment.

### Teaching of diagnostics and therapy

Every student performed the following steps three times with the respective simulator: step 1) use of vaginal speculum (Orchid Wide SX, Bridea Medical, Amsterdam, Netherlands) with insertion into artificial vagina, step 2) use of colposcope for all following steps, step 3) Pap-smear with separate ecto- and endocervical brush, step 4) application of acetic acid and Lugol’s iodine, step 5) cervical biopsy of areas suspicious for precancerous lesions, step 6) endocervical curettage and step 7) application of ferric sulfate for hemostasis. For each of the described training cycles, the students received a new artificial cervix for their simulator.

After this diagnostics-simulation, the students proceeded to the electrosurgical excision. The used generator for the excisions was the product VIO 300D® and the neutral electrodes were NESSY® Omega Plate (both Erbe Elektromedizin GmbH, Tübingen, Germany). The students were offered 10-, 15-, 20- and 25-mm-diameter loops (Wolfram Schlingenelektrode, Erbe Elektromedizin GmbH, Tübingen, Germany) and chose their electrosurgical loop by themselves according to their own estimation, after having inspected the artificial cervix to be operated. All students had been taught about the definition of the LEEP-score and how a “perfect” cone should be formed following Takacs et al. [31, 32]. During the excision, the teacher assisted the students by preparing the simulators but did not give any surgical advice to any of the students. After finishing the excision, the students could use a ball electrode for simulating hemostasis. The artificial cervix was then replaced and the students repeated the described process until five electrosurgical excisions had been performed. All artificial cervices were standardized, showing an equal size and shape, although it must be noticed, that the shape of the sausages in the conventional simulator (Group A) was not always exactly the same. All cervices were placed 10 cm deep into the artificial vagina. Furthermore, all artificial acetic white stains were placed at three o’clock from the cervical os and had a diameter of approximately five millimeters.

### Methods for objective evaluation

After the excision, the removed artificial tissue was measured according to the LEEP-Score of Takacs et al. [31, 32]. Subsequently, the students could decide, if another excision should be performed, in order to reach the desired cone depth, if this had not been achieved by the first excision. The best possible LEEP-Score a student could achieve was an adequate cone depth (8-10 mm) with one single excision. In such a case, the student received 0 points. A cone depth of 11 mm would be 1 mm to deep and cone depth of 7 mm would be 1 mm too shallow. With every mm of deviation of the desired cone depth (cone to deep or too shallow) and with every additional excision, an extra penalty point was awarded. For instance, a student, who generated a cone depth of 6 mm could decide, if he or she wanted to accept this result (LEEP-Score: two, because 2 mm too shallow) or if he or she wanted to execute another excision in order to improve the cone depth. If the second excision generated e.g. 2 mm of removed tissue, adding to a cone depth of all in all 8 mm (6 mm in first and 2 mm in second excision) the LEEP-Score was one, as the desired cone depth of 8–10 mm was reached, but with one additional excision.

The teacher noted the LEEP-Score as well as the macroscopic resection-status (R0 or R1). The latter was done by inspecting the resected cone. If all of the artificial acetic white stains were removed with the first excision, a macroscopic R0-resection was documented. If white stains remained on the cervix, a macroscopic R1-resection was stated. Furthermore, the digital camera of the colposcope allowed the teacher to note the amount of contacts between loop and artificial vagina and / or speculum by visual inspection on a separate monitor.

### Methods for subjective evaluation

After the training, both groups then assessed the seminar and their simulator with 18 questions. The responses were given on Likert-scales raging from 1 to 10 with 1 equaling “strongly agree” or “very good” and 10 equaling “strongly disagree” or “very bad” according the validated evaluation-form of Takacs et al. [31, 32]. Group B additionally assessed the novel simulator with four questions, again with Likert-scales (1–10). In order to prevent any disadvantage, the respective other simulator was demonstrated to all students and they were given an opportunity and actively encouraged to work with it after finishing the evaluation of the seminar.

### Statistics

Statistical analysis was done by the program R Core Team, version 2020 (R Foundation for Statistical Computing, Vienna, Austria). The significance level (p) was 0.050. To compare whether the difference between Group A and B was statistically significant concerning the 18 questions answered on the Likert scales, as well as the differences between the change of the LEEP-scores [31, 32], the Mann-Whitney-U-Test was used.

## Results

Both simulators appeared to be of aid for the training of diagnostics and treatment of cervical precancerous lesions. The novel simulator showed several advantages compared to the conventional model.

### Subjective assessment of Group A and B

**Fig. 2:**
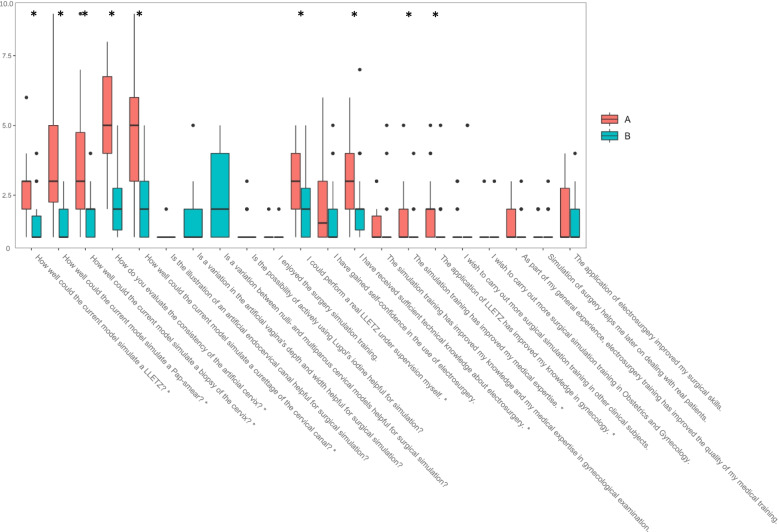
Subjective assessment of novel and conventional simulator by medical students: Boxplots visualizing the mean score of all answers to the evaluation-form. The boxes indicate the interquartile range and the black bar in the middle of each box shows the median. The whiskers stand for minimum and maximum point scores. The dots depict the outliers. Orange bars refer to Group A (training with conventional simulator) and turquoise bars refer to Group B (training with novel simulator). Those questions only answered by Group B only show one boxplot each. * symbolize statistically significant differences between Group A and B

Eighteen questions (Likert scale 1–10, 1 = strongly agree / very good, 10 = strongly disagree / very bad) focused on the assessment of the seminar and the simulators by the students. Nine of these 18 questions showed statistically significant differences favoring Group B: The question “How well could the current model simulate a LLETZ?” was answered by Group A with a mean point score of 2.9 and by Group B with 1.4 (*p* < 0.001). Group A rated the question “How well could the current model simulate a Pap-smear?” with a mean of 3.7 and by Group B with a mean of 1.4 points (*p* < 0.001). The question “How well could the current model simulate a biopsy of the cervix?” was rated with a mean of 3.5 points by Group A and 1.8 points by Group B (*p* < 0.001). The question “How well could the current model simulate a curettage of the cervical canal?” scored a mean of 4.8 points in Group A and a mean of 2.2 points in Group B (*p* < 0.001). Group A answered the question “How do you evaluate the consistency of the artificial cervix?” with a mean of 5.3 and Group B with a mean of 2.2 points (*p* < 0.001). The question “I could perform a real LLETZ under supervision myself” was rated with a mean of 3.0 points by Group A und 1.9 points by Group B (*p* < 0.001). The question “I have received sufficient technical knowledge about electrosurgery” received a mean of 2.9 points by Group A and a mean of 2.2 points by Group B (*p* < 0.02). Group A and B rated the question “The simulation training has improved my medical expertise” with a mean of 1.7 and 1.2 points, respectively (*p* < 0.004). Finally, the question “The application of LLETZ has improved my knowledge in gynecology” received a mean of 1.9 points from Group A and a mean of 1.3 points from Group B (*p* < 0.007). These findings are visualized in Table 1 and Fig. 2. The other questions showed no statistically significant difference between the answers in Group A and B and are depicted in the Additional file 1 of this work.

**Table 1:** Comparison of subjective assessment of conventional simulator (Group A) and novel simulator (Group B) using Likert scales (1–10, 1 = strongly agree / very good, 10 = strongly disagree / very bad)

Questions:	Group A (conventional simulator)	Group B (novel simulator)	*P*-value
How well could the current model simulate a LLETZ?	2.9	1.4	*p* < 0.001
How well could the current model simulate a Pap-smear?	3.7	1.4	*p* < 0.001
How well could the current model simulate a biopsy of the cervix?	3.5	1.8	*p* < 0.001
How well could the current model simulate a curettage of the cervical canal?	4.8	2.2	*p* < 0.001
How do you evaluate the consistency of the artificial cervix?	5.3	2.2	*p* < 0.001
I could perform a real LLETZ under supervision myself	3.0	1.9	*p* < 0.001
I have received sufficient technical knowledge about electrosurgery	2.9	2.2	*p* < 0.02
The simulation training has improved my medical expertise	1.7	1.2	*p* < 0.004
The application of LLETZ has improved my knowledge in gynecology	1.9	1.3	*p* < 0.007

### Additional assessment of Group B

Group B additionally assessed certain aspects and components of the novel simulator with four questions, again with Likert-scales (1–10, 1 = strongly agree / very good, 10 = strongly disagree / very bad). Students from Group B rated the question “Is the illustration of an artificial endocervical canal helpful for surgical simulation?” with a mean of 1.03 points. The question “Is a variation in the artificial vagina’s depth and width helpful for surgical simulation?” was answered with a mean of 1.6 points. The question “Is a variation between nulli- and multiparous cervical models helpful for surgical simulation?” received a mean of 2.5 points. The last question solely for Group B, asking if the possibility of actively using Lugol’s iodine was helpful for simulation, was rated with a mean of 1.2 points. These findings are depicted in Table 2.

**Table 2:** Additional assessment of Group B using Likert scales (1–10, 1 = strongly agree / very good, 10 = strongly disagree / very bad)

Questions:	Group B (novel simulator):
Is the illustration of an artificial endocervical canal helpful for surgical simulation?	1.03
Is a variation in the artificial vagina’s depth and width helpful for surgical simulation?	1.6
Is a variation between nulli- and multiparous cervical models helpful for surgical simulation?	2.5
Is the possibility of actively using Lugol’s iodine helpful for simulation?	1.2

### LEEP-Score

**Fig. 3:**
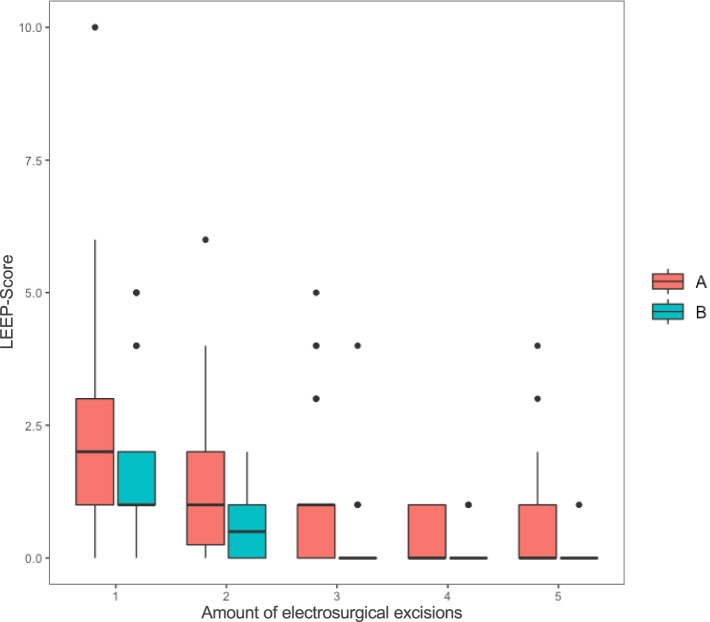
Learning curves: Boxplots visualizing the mean LEEP-Score of Group A and B during their five electrosurgical excision. The boxes indicate the interquartile range and the black bar in the middle of each box shows the median. The whiskers stand for minimum and maximum point scores. The dots depict the outliers. Orange bars refer to Group A (training with conventional simulator) and turquoise bars refer to Group B (training with novel simulator)

**Fig. 4:**
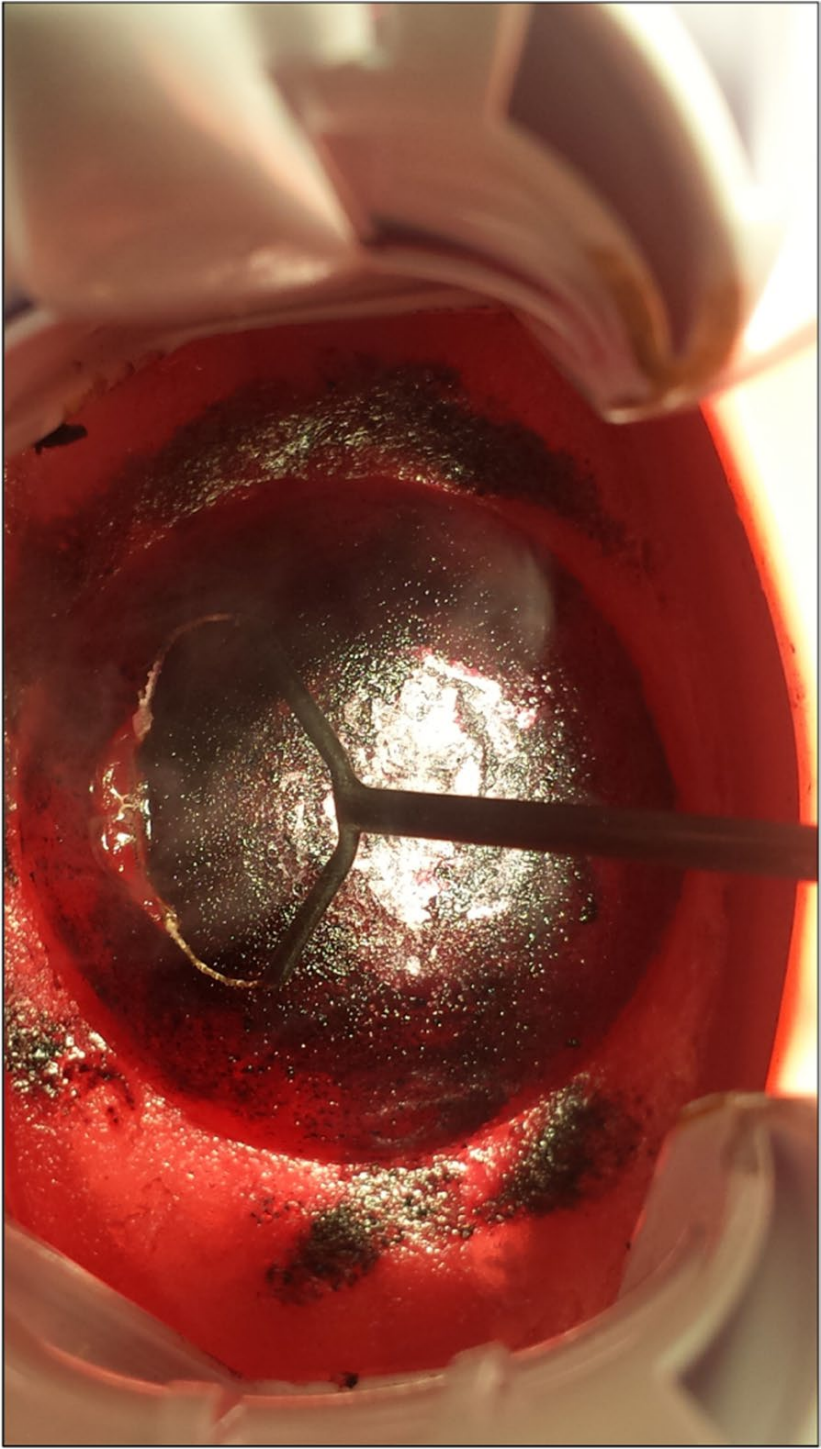
The novel simulator depicting the surgical loop (Erbe Elektromedizin GmbH, Tübingen, Germany), vagina, cervix with acetic white stains, and speculum during electrosurgical excision after having applied Lugol’s iodine

In order to objectively compare the training results between Group A and B, the change of LEEP-Scores [31, 32] throughout the five excisions every student generated were compared. The best possible LEEP-Score is 0. The higher the LEEP-Score, the less desirable the quality of the excison’s result. Both simulators generated an evident learning curve. Students in Group A achieved a LEEP-Score with a mean of 2.1 and in Group B of 1.4 in their first excision. In their fifth excision, Group A generated a mean LEEP-Score of 0.7 and Group B of 0.03. Yet, these differences were not statistically significant (*p* < 0,647). The learning curves of both groups are visualized in Fig. 3. Figure 4 displays an electrosurgical excision with the novel simulator.

### Contacts between electrosurgical loop and vagina or speculum as well as resection status

In addition to the LEEP-Score, the amount of contacts between the electrosurgical loop and the simulator’s artificial vagina as well as the speculum during each excision were counted. In reality, such contacts lead to injury due to incision or burning and can cause serious complications [26–30]. In Group A, 18.7% of all excisions contained a contact between the loop and the simulator’s artificial vagina. 12.7% lead to an additional contact with the speculum. In Group B, 14.0% of the excisions showed the loop touching the artificial vagina and 1.3% showed the loop touching the speculum. Moreover, the amount of macroscopic R0- and R1-resections was compared between both groups. 22.7% of the resections in Group A led to a macroscopic R1-resection. In Group B, this was the case in 13.3%.

## Discussion

In congruence to our prior findings [42], the novel simulator for training diagnostics and therapy of precancerous cervical lesions showed several aspects of superiority compared to the conventional simulator, suggesting it is a valid alternative.

The novel simulator’s potential is highlighted firstly by the subjective assessment of the seminar. Students in Group B (novel simulator) rated pivotal practical aspects such as the simulation of a LLETZ, a Pap-smear, a cervical biopsy and cervical curettage better than Group A with the conventional simulator. Moreover, the mimicking of a human cervix was also seen as more realistic in the novel simulator, with all of the mentioned differences between Group A and B showing statistical significance (*p* < 0.001). In addition to these practical aspects, students in Group B felt significantly better educated and prepared for real surgery, stating a higher confidence to perform a real LLETZ themselves (*p* < 0.001). Adding to this, Group B subjectively felt a higher degree of technical knowledge about electrosurgery (*p* < 0.02) and improved medical expertise in general (*p* < 0.004) as well as expertise in gynecological examination in particular (*p* < 0.007) than students in Group A. Whether this subjective feeling translates to actual improved competence gain for Group B was not evaluated in the scope of the study. Additionally, Group B rated all selected aspects of the novel simulator including the illustration of an artificial endocervical canal (1.03 points), a variation in the artificial vagina’s depth and width (1.6) as well as a variation between nulli- and multiparous cervical models (2.5) and the possibility of actively using Lugol’s iodine (1.2 points) as helpful. However, it should be noted that the key differences between the simulators were given by the question phrasing and it is not known whether students would have identified and appreciated these differences on their own.

The LEEP-Score had already been evaluated by Takacs et al. [31, 32], providing a valid method for measuring the learning curve in Group A and B. The fact that these learning curves were relatively similar can be seen as proof of concept for the novel simulator. It is not surprising, that differences between the learning curves favoring Group B were not statistically significant, as the conventional simulator had already proven that it can provide ample training options and valid data. Higher case numbers could possibly show a statistically significant difference in the LEEP-Scores between the two simulators, which could be subject of future studies.

The observation of less contacts between loop and vagina or speculum and a higher rate of macroscopic R0-resections in the novel simulator could be due to the different vaginal shapes. Since the conventional simulator’s vagina was mimicked by a round drain pipe, the speculum could not be opened as wide as it could in the novel simulator, making it less realistic and potentially more likely to touch with the electrosurgical loop during an excision. This can be seen as an advantage of the novel simulator against its conventional counterpart, as it apparently enables easier training and is more similar to a real vagina. Yet, this could also be seen as advantage of the conventional simulator, since it offers a possibly more challenging training with potentially more thorough preparation for real surgery. More importantly, this finding emphasizes the risk of patient injury during electrosurgical excisions especially with novice surgeons and therefore highlights the importance of adequate training before performing surgery on patients.

The participating students in part criticized the consistency of both the conventional but also the novel artificial cervix as being too soft. This could be improved in studies to come. As limitation of this study, it must be stated, that medical students, with only limited familiarity with real life reproductive anatomy and who had never before performed an electrosurgical excision of the cervix, could only partly answer questions such as “How well could the current model simulate a LLETZ?”. It would have been beneficial to add a group of experienced Gynecologists with specialization in colposcopy and electrosurgery to evaluate and compare both simulators as well. This should be further evaluated in future studies.

## Conclusion

The described novel simulator offers a valid training option with several advantages compared to conventional simulators. This contributes to the education of future health care providers in this significant field of clinical diagnostics and therapy, offering a possibility for standardized high quality education. Further studies are required, in order to further establish modern aspects of simulation of Gynecologic interventions and thus supporting patient safety.

## Supplementary Information

**Additional file 1.**

## Data Availability

The datasets used and analysed during the current study are available from the corresponding author on reasonable request.

## Abbreviations

LEEP: Loop Electrosurgical Excision Procedure
LLETZ: Large Loop Excision of the Transformation Zone
2D: Two dimensional
3D: Three dimensional
Pap-Smear: Papanicolaou-Smear
