# Supplementary material for: Evaluating a novel 3D printed model for simulating Large Loop Excision of the Transformation Zone (LLETZ)
Source: 3D Print Med. 2022 Jun 8;8:15. doi: 10.1186/s41205-022-00143-x (PMC9175315; doi:10.1186/s41205-022-00143-x)
Supplement: Supplementary file 1 — Additional file 1. [file 41205_2022_143_MOESM1_ESM.docx]

# Additional file 1

The following questions or statements were part of the evaluation-form, which was answered after the seminar by the students of both Group A and B. The showed no statistically significant differences between the two groups. They were answered with Likert-scales, ranging from 1 to 1, with 1 = strongly agree / very good, 10 = strongly disagree / very bad.

- I enjoyed the surgery simulation training.
- I have gained self-confidence in the use of electrosurgery.
- The simulation training has improved my knowledge and my medical expertise in gynecological examination.
- I wish to carry out more surgical simulation training in other clinical subjects.
- I wish to carry out more surgical simulation training in Obstetrics and Gynecology.
- As part of my general experience, electrosurgery training has improved the quality of my medical training.
- Simulation of surgery helps me later on dealing with real patients.
- The application of electrosurgery improved my surgical skills.
- The simulation training has improved my knowledge and my medical expertise in gynecological examination
